# From violent lone-actor types to lone-actor grievance-fueled violence: The Aarhus University shooting as an example of multi-facetted offender motivations and context-sensitive boundaries between violent lone-actor categories

**DOI:** 10.3389/fpsyg.2022.995818

**Published:** 2023-01-17

**Authors:** Christopher Kehlet Ebbrecht, Lasse Lindekilde

**Affiliations:** ^1^Department of Psychology and Behavioural Sciences, School of Business and Social Sciences, Aarhus University, Aarhus, Denmark; ^2^Department of Political Science, School of Business and Social Sciences, Aarhus University, Aarhus, Denmark

**Keywords:** lone-actor grievance-fueled violence, school shooting, incels, radicalization, violent extremism

## Abstract

Over the last decade, western societies have experienced an increase in acts of mass violence carried out by lone actors. While this concept is mostly associated with lone-actor terrorists, it also involves the actions of other single perpetrators, e.g., school shooters, workplace attackers, rampage shooters, and some forms of incel violence. In this article, we argue in favor of moving away from such categorization of violent lone-actor types and toward the unifying concept of lone-actor grievance-fueled violence. We illustrate the analytical benefits gained from such a conceptual shift by analyzing the Danish Aarhus University Shooting in 1994, where a single offender killed two students. While this attack is widely accepted as the only Danish school shooting in history, we identify signs of an extremist misogynist worldview held by what we today would call incels. This case serves as an illustration of the blurred and context-sensitive boundaries between violent lone-actor types and how nuances in offender motivation can be lost when lone-actor attacks are classified within a typological framework. Rather than simply recasting the Aarhus University Shooting as an incel attack considering the recent development of this category, we argue for the need to embrace the conceptualization of lone-actor grievance-fueled violence, which points toward the common genesis of lone-actor violence and allows for multi-faceted offender motivations. Using the Aarhus University shooting as a steppingstone, we discuss the pitfalls of lone-actor violence typologies and the advantages of the unifying lone-actor grievance-fueled violence conceptualization for both academia and practice.

## Introduction

Over the last decade, western societies have experienced an increase in acts of mass violence carried out by lone actors (Hamm and Spaaij, 2017; Kenyon et al., 2021), that is, a perpetrator who single-handedly executes an attack, has no direct affiliation with an extremist group or terrorist organization, or is under direct command or influence of a terrorist leader or group (Lindekilde et al., 2017). While the notion of lone actors is mostly associated with lone-actor terrorists, the definition also applies to other single offenders, e.g., school shooters, workplace attackers, rampage shooters, fame-seeking mass shooters, mass murderers, and some forms of incel violence (Hempel et al., 2000; Langman, 2013; Lankford, 2013; Capellan and Anisin, 2018; Taylor, 2018; Capellan et al., 2019; Silva and Greene-Colozzi, 2019; Hoffman et al., 2020; Clemmow et al., 2020b). Historically, lone-actor violence has been considered a particularly challenging phenomenon to prevent due to their relatively more solitary and unpredictable radicalization trajectory (Bakker and de Graaf, 2010; Alakoc, 2017).

In this article, we argue in favor of moving away from a distinctive typology of violent lone-actor types and toward the unifying conceptualization of lone-actor grievance-fueled violence, which suggests that these seemingly different offenders may share a common genesis (McCauley et al., 2013; Barry-Walsh et al., 2020; Clemmow et al., 2020b). Despite increasing usage of the concept, the literature still seems to revolve in large part on the typological approach (e.g., Lankford, 2013; Capellan et al., 2019). To illustrate the analytical benefits of embracing the lone-actor grievance-fueled violence concept, we analyze the Aarhus University Shooting in 1994, where a single offender killed two students and wounded another two. While this attack is widely accepted as the only Danish school shooting in history, we, using restricted case material, identify several signs of an extremist misogynist worldview held by what we would call incels today. Although case files clearly show that police officers investigating the attack were aware of a potential misogynistic motivation for the attack, the case has been reduced in journalistic writing and public memory to an example of a school shooting. To illustrate, both the Danish and English Wikipedia pages for the attack (visited January 11, 2022) classify it as a school shooting and make no mention of the victims’ gender or the offender’s motivation. We argue that this reduction of complexity and neat categorization partly reflects the fact that while at the time of the attack, bullying and frustrated academic performance were well known motivations for school shootings, what we today refer to as inceldom had not yet been conceptualized and described as a motivation for lone-actor violent attacks. Hence, the Aarhus University Shooting is an example of the blurred and context-sensitive boundaries between lone-actor types and shows how nuances in offender motivation can be lost when lone-actor attacks are classified within a typological framework. However, rather than simply recasting the Aarhus University shooting as an incel attack in light of the recent development of this category, we argue for the need to embrace the conceptualization of lone-actor grievance-fueled violence, which points toward the common genesis of lone-actor violence and better allows for multi-faceted offender motivations. Although a categorical approach to understanding lone-actor violence has its merits and may be valuable in addressing certain research questions, we contend that the lone-actor grievance-fueled violence framework holds potential for a more comprehensive theory of lone-actor violence.

In the following, we first review the academic literature on violent lone-actor types and show how conceptual tensions can be resolved and subsumed under the heading of “lone-actor grievance-fueled violence.” Building on this, we then analyze the Aarhus University shooting with a focus on perpetrator motivation. We use the case as a prism to exemplify the blurred and context-sensitive boundaries between violent lone-actor types and illustrate the advantages of a more unifying conceptual framework. The analysis rests on flexible coding (Deterding and Waters, 2021) of restricted case documents, including forensic reports, witness testimonies, testimonies of friends, family and university teachers/supervisors as well as the perpetrator’s diaries, personal notes etc. We conclude the article by discussing what the case is a case of and use this as a steppingstone to discuss the pitfalls of lone-actor violence typologies. We argue that the key propositions of the unifying lone-actor grievance-fueled violence perspective is a deepened understanding of the motivational impetus behind violence carried out by single perpetrators, which allows for multi-facetted motivations and makes assessment of motivations less dependent on context and time-dependent categories. We contend that such a perspective carries crucial implications for the practical prevention of mass violence.

### From violent lone-actor “types” to lone-actor grievance-fueled violence

While the concept of “lone actors” is quite new within academia and in layman’s terms, the phenomenon of individuals who single-handedly commit acts of mass violence is not. As far back as the 14th century, there are reports of cases of “amok” or “beserk” the Malaysian notions for individuals who exhibit “sudden unprovoked outbursts of uncontrollable behavior, often leading to indiscriminate injury or death to others” (Hempel et al., 2000, p. 582). During the 1900s, such cases were, in a western context, named “sudden mass assault by a single individual” (SMASI), which is equivalent to what we refer to in this article as violence committed by a lone actor. Specifically, we define lone actors as individuals who operate as a single perpetrator in the execution of an attack without direct affiliation with an extremist group, network, or organization, without following direct commands of, or being under direct influence of, a leader figure, group, or otherwise likeminded individuals (Lindekilde et al., 2017, p. 1). Moreover, we only pay attention to perpetrators of mass violence carried out on a public stage in one, or multiple closely related, location(s) within a short period of time (e.g., Capellan et al., 2019, p. 814). We thus distinguish lone actors from other single offenders committing violence resulting in death, e.g., homicide, familicide, serial killings, crime-related killings etc. (Fridel, 2017; Liem et al., 2018a, 2018b; Frei and Illic, 2020).

The literature highlights at least five seemingly different violent lone actor types. First, there is the lone-actor terrorist (once known as the lone-wolf terrorist), who acts out of radical ideological, be it politically right-wing, left-wing, nationalist, single-issue or religious extremism (Spaaij, 2012; Gill et al., 2014; Doosje et al., 2016; Kenyon et al., 2021). Second, and perhaps best known, school shooters attack their current or former educational institution (e.g., Leary et al., 2003). Similarly, the third category, workplace attackers, take violent action against their current or former workplace (Lankford, 2013). Fourth, there is the recent classification of incels (involuntary celibates), who are motivated by a specific worldview (Baele et al., 2019; Hoffman et al., 2020) stemming from misogynistic attitudes, which has found to be a driver of other forms of sexual violence and death (e.g., Willmott et al., 2017; Sowersby et al., 2022). While it can be argued that incels could represent a subgroup of lone-actor terrorists due to the seemingly overlapping ideological motivation, the literature tends to treat them as different types, at least for now (Ebbrecht, 2022). The final group is often referred to as “rampage shooters” or simply “mass murderers” and essentially represent an “other” category as they do not match any of the preceding lone-actor categories (Capellan et al., 2019). This categorical approach to lone actors is not only predominates the academic field, but is also widely used within both intelligence services (e.g., Jah and Khoshnood, 2019; Centre for Terror Analysis, 2022), legislation and criminal law (Retsinformation, 2019), and social media (e.g., Elamroussi et al., 2022; Hamilton, 2022; Wikipedia, 2022).

In this paper, we argue that classifying lone actors within a typological framework like the one above entails a risk of overlooking the multi-facetted motivational impetus for engaging in mass violence, which may be more similar than different across offender types. Importantly, our quarrel is not with lone-actor typologies *per se*. Indeed, the literature has several such useful frameworks. For instance, using two different samples (*n* = 10, *n* = 29) of US school shooters, Langman (2009, 2013) provided evidence that offenders could be meaningfully differentiated into (1) traumatized shooters who come from broken homes where they have experienced physical and/or sexual abuse; (2) psychotic shooters who showed symptoms of either schizophrenia or schizotypal personality disorder; and (3) psychopathic shooters demonstrating narcissism, lack of empathy and conscience, sadistic tendencies, etc. Similarly, Ioannou et al. (2015) differentiated school shooter characteristics into (1) disturbed, (2) rejected, and (3) criminal offender categories. Peter et al. (2019) similarly disaggregates a sample of 44 German whereas clusters in relation to situational factors of “rampage shooters” into (1) narcissistic offenders suffering from addiction or affective disorder, (2) psychotic offenders, and (3) aggressive, narcissistic, or anxious offenders who often have an affective disorder. Comparing lone-actor terrorists and mass murderers, Clemmow et al. (2020b) identifies different clusters when comparing offenders in relation to different types of risk factors (see also Gruenewald et al., 2013). For instance, when looking at propensity factors, perpetrators can be categorized as either “criminal,” “stable” or “unstable,” whereas clusters in relation to situational factors are labeled “low stress,” “high stress (social),” and “high stress (interpersonal).” These detailed typologies are all valuable in the sense that they 1) illustrate the principle of equifinality in (lone-actor) radicalization, where no single offender profile exists, and where the relative causal weight of similar risk factors may differ across cases (Borum, 2011; Gill et al., 2020, 2021), and 2) differentiate selected violent lone actors on the basis of a specific research question (e.g., mental health characteristics, radicalization trajectory, etc.). However, it is our contention, that the five broad lone-actor types we address in this paper and the way they are used in literature are more problematic for two reasons.

First, as noted by others, distinguishing between lone-actor terrorists, workplace attackers, school shooters, rampage shooters, and violent incels runs the risk of overlooking commonalities between these categories. Already in McCauley et al. (2013) argued that the similar mechanisms of radicalization among lone-actor terrorists and school shooters suggest that these offenders more of less follow the same radicalization trajectory. These findings have later been supported by, e.g., Lankford (2013), Capellan (2015), Capellan et al. (2019), who all provide evidence that different types of lone actors develop through similar social and psychological processes. Finally, while not making an actual comparison between offender types, a recent systematic review on risk factors and mechanisms of radicalization of all five lone-actor types mentioned in this paper finds that many of the same factors are present across offender categories (Ebbrecht, 2022). On this evidence basis, it can thus be questioned whether relying on a typological framework revolving around these five violent lone-actor types is the best way forward. Again, this is not to say that a typological framework *per se* is undesirable, but rather to question whether the current broad classification cuts the cloth where it should.

Second, and of key importance to our argument, we argue that the distinction between lone-actor terrorists, workplace attackers, school shooters, rampage shooters, and violent incels does not capture the motivational impetus behind the form of mass violence these offenders engage in. While we do not suggest that these classifications have developed out of a wish to disseminate offender motivation, as has been the case in the literature in differentiation adolescent violent offenders (Filkin et al., 2022) and rioters (Willmot and Ioannou, 2017), we argue that the typological framework implies a single specific motivational impetus for each lone-actor category; i.e., lone-actor terrorists are motivated by extremist ideology, school shooters and workplace attackers by interpersonal conflict in educational or workplace settings, violent incels by misogynist beliefs and a desire for revenge against women, and rampage shooters by reasons still unknown. It is our contention that highlighting these characteristics of lone actors as different motivational impetuses for mass violence essentially reflects others’ attribution of behavior rather than actual disentanglement of offender motivation. In other words, the presumed motivations mentioned above reflect how others explain the actions of lone actors rather than answer why they actually commit acts of violence. Our purpose is thus not to ignore or deny the unquestionably different features of some lone-actor perpetrators but rather to stress that these features are different concrete manifestations of the generic motivational impetus underlying lone-actor grievance-fueled violence. As the literature clearly illustrates, these presumed motivations do not always fit the specifics of a particular lone-actor attack. Therefore, we argue that an important aspect of uncovering the motivational impetus behind mass violence perpetrated by lone offenders is to remain open to the particular and multiple offender motivations that might be at play; something we believe is achieved with the concept of lone-actor grievance-fueled violence.

The key proposition of this recent conceptual framework is to focus on the mechanisms of grievances, that is, a perceived sense of injustice or humiliation that fosters feelings of anger, hatred and a desire for revenge against the alleged perpetrators of wrong (McCauley and Moskalenko, 2017). Grievances can be *personal*, meaning that the perceived injustice is done to the offender personally, or they can be *group-based* (or *political*), i.e., the lone actor is moved to violent action in response to political events or trends hurting their perceived social or political group (McCauley and Moskalenko, 2008). For instance, some studies find that lone-actor terrorists often hold political grievances and are far more prone to take violent action on this basis compared to other offender types (Gill et al., 2014; Capellan et al., 2019). In contrast, school shooters are often driven to violence by personal grievances, often due to experiences of bullying and other varieties of interpersonal rejection, isolation and conflict (Leary et al., 2003; Sommer et al., 2014; Kohlbeck and Nelson, 2020). However, such findings are not consistent. Both Capellan (2015) and Capellan and Anisin (2018) find that even what they classify as “ideological shooters” are in fact not always motivated by ideological grievances. Likewise, Malkki (2014) identifies political elements in the writings of several school shooters.

The literature thus seems to be at an impasse where the motivation behind lone-actor violence best can be regarded as a combination of political grievances and personal vendettas (Spaaij, 2010; Altay et al., 2020; Kenyon et al., 2021). It is by taking these inconsistencies seriously, that lone-actor grievance-fueled violence developed as a concept (Capellan, 2015; Capellan et al., 2019; Clemmow et al., 2020b), making the fundamental proposition that seemingly different lone actors may radicalize through similar social and psychological processes and thus share a common genesis with grievances as the key denominator. Here lies the potential for a unifying perspective and more comprehensive theorization of lone-actor violence. Importantly, this perspective does not propose that experiences of injustice or humiliation alone lead to lone-actor violence (Clemmow et al., 2020a, 2020b; Kenyon et al., 2021; Ebbrecht, 2022). If this was the case, the phenomenon would be much more widespread. For example, while many men may experience humiliation because they cannot find romantic partners and are rejected by women, few develop extreme misogynic views and justify violence against women as vengeance for this grievance. Other factors are at play. Research suggests that personal characteristics also play a role in determining violent outcomes in general and lone-actor violence in particular (Gøtzsche-Astrup and Lindekilde, 2019). Experiences of humiliation may appear much more acute to individuals who show signs of narcissism and grandiose entitlement. If your need for status is very strong, humiliation-infused grievances would appear all the more agonizing and in need of repair (Storr, 2021, p. 69).

As the typological approach entails crucial limitations in the understanding of lone-actor motivation and thus has practical implications for prevention of grievance-fueled violence, we argue that the time has come to move away from a categorical framework to the more unifying theory of lone-actor grievance-fueled violence. To illustrate the analytical benefits of doing so, we show how the attacker behind the Aarhus University shooting, described and categorized as the only Danish school shooting, showed signs of motivation that match both the school shooter and the violent incel lone-actor categories. The case analysis shows how the boundaries between these categories are blurred in practice and how case categorization of motivations is context-sensitive, dependent on the available and known categories at a given point in time and space. Abandoning a categorical approach to understanding this case and being open to the particular motivations at play has the benefit of uncovering multiple and interwoven offender motivations and thus reveals a new layer of complexity to be taken into account in the development of interventions to prevent lone-actor violence.

## Materials and methods

The following analysis offers a case study of the Aarhus University Shooting. From June 2021 to March 2022, we collected data from several open and closed secondary sources using a snow-balling strategy. The materials include restricted police reports, investigative interviews with key witnesses, family members and acquaintances of the offender, transcripts from a TV documentary and a journalistic podcast about the case, and newspaper articles. We also conducted one interview with two of the leading police investigators of the attack. All materials quoted in the article are listed in Table 1.

**Table 1:** List of quoted materials.

Reference	Material	Data type
Danish National Archives (1994a)	Report of crime	Closed source
Danish National Archives (1994b)	First police interrogation of the offender’s thesis supervisor	Closed source
Danish National Archives (1994c)	First police interrogation of the offender’s brother	Closed source
Danish National Archives (1994d)	Suicide letter written by the offender	Closed source
Danish National Archives (1994e)	Notes from police search of the offender’s apartment	Closed source
Danish National Archives (1994f)	Police interrogation of friend of the offender	Closed source
Danish National Archives (1994g)	Police report on search of the offender’s apartment	Closed source
Danish National Archives (1994h)	Police interrogation of the offender’s father	Closed source
Danish National Archives (1994i)	Second police interrogation of the offender’s brother	Closed source
Danish National Archives (1994j)	Second police interrogation of the offender’s thesis supervisor	Closed source
Danish National Archives (1994k)	Correspondence between offender and thesis supervisor	Closed source
Fisker (2003)	TV documentary	Open source
Bekholm and Hansen (2019)	Journalistic podcast	Open source

We are the first researchers to obtain full access from the investigating police force to all police files on the case. The police files contain more than 2000 pages of material, including investigative interviews with key witnesses and bystanders with a close relation to the offender, e.g., family members, acquaintances of the offender, and professors he was in contact with at university; search reports from the offender’s home containing copies of diary entries, notebooks, calendar markings, correspondences etc.; the offender’s suicide note; reports from the first police officers and responders at the crime scene; forensic and autopsy reports; crime scene photos and much more. Due to the sensitivity of this material, it was only accessible at the archival site, and photocopying of material was not allowed. Quotes and descriptions provided in the analysis below are therefore based on handwritten citations and notes from the archive. Out of ethical considerations, we have not attempted to contact the offender’s living relatives for material (e.g., the offenders’ full diary, which has been returned by the police) that could shed further light on the case.

In addition to the restricted police files, we rely on two types of open-source data. First, we accessed and read the Danish media coverage of the incident at the time of the attack and the following years. Second, we rely on transcripts of a TV documentary about the case made in 2003 for national TV, and a journalistic podcast about the case made in 2019 for a local Aarhus newspaper. These sources are interesting because the journalists behind both managed to find and interview people who lived at the same college dorm as the offender at the time of the attack. The documentary and podcast thus contain new material that is not in the police files. We interviewed the journalist behind the podcast to further probe this new material.

Finally, after examining the police files and the open-source data, we interviewed two of the leading police investigators, who were young detectives at the time of the attack. One is retired, and the other is a senior police officer. While the interviewees’ memory of details of the case is naturally fading, it was clear that the Aarhus University shooting had left a lasting impact on the detectives and was a case they had discussed many times since. The interview served as a validation of our understanding of the case material and as an opportunity to discuss the offender’s motivations.

### Data analysis

To analyze the large amount of closed and open-source data, we followed the guidelines of flexible coding (Deterding and Waters, 2021) in three stages. First, to gain a sense of the bigger picture of the case, we used index codes (i.e., coding of large chunks of text) to reduce data. Index codes were generated *in vivo* and revolved around major themes that emerged in the data. In line with the flexible coding methods, we especially made a note of chunks of text in the data that were “particularly concise, articulate, or poignant” (Deterding and Waters, 2021, p. 727). Second, we applied the following analytic codes in the form of known risk factors of lone-actor radicalization (Kenyon et al., 2021; Ebbrecht, 2022) whenever they presented themselves in data: sociodemographic background, ties to social networks, interpersonal rejection, mental health, subclinical personality traits, strain, grievances, emotional traits and states, and cognitive content and processes. Third, to ensure theoretical validity of our analysis, we analyzed how our analytical codes were related to each other to form a coherent developmental trajectory of the offender.

## Analysis: The Aarhus University shooting

We begin the analysis with a description of the attack at Aarhus University in 1994. Next, we highlight two simultaneously present grievances and show that the offender indeed had multi-facetted motivations for engaging in mass violence. To illustrate how grievances alone not are the sole driver of radicalization, we delve further into the offender’s life experiences in childhood, adolescence, and adulthood at university to identify other risk factors. We then tentatively propose that these experiences manifested themselves as a personality structure revolving around grandiose entitlement, which was a crucial factor in the radicalization of the offender. Finally, we show how the offender ruminated about violence, and how the onset of mental health issues and sudden mental strain might have influenced the timing of the attack. We end the analysis with a summary considering the interplay between the identified risk factors in motivating violent action.

### Case description of the attack

On April 5, 1994, the first day after the Danish Easter holidays, the offender, a male student, enters the building at the corner of Niels Juels Gade and Aldersrovej that houses the Department of Nordic Studies at Aarhus University (Bekholm and Hansen, 2019). Wearing a blue jacket, yellow shirt, patterned tie, and carrying a blue sports bag, he walks around the hallways before going down to the basement and entering the cafeteria filled with over a 100 students (Fisker, 2003). He sits there for a couple of minutes and then leaves only to return moments later. After a small argument with the cafeteria staff, he sits down at a table in the non-smoking area and waits. Around 11 a.m., people start going to lectures and exams, and at 11:15, the cafeteria is empty except for a few students sitting at different tables scattered across the room. The offender gets up from his chair and resolutely walks toward a table with two female students. About three meters away from them, he stops, pulls out a sawed-off shotgun from his sports bag, takes aim and yells: “I hate you!” He fires two shots, instantly killing one of the students and mortally wounding the other. He turns to a third woman sitting at another table close by, starts walking toward her, but then stops to reload the shotgun (Bekholm and Hansen, 2019). The woman throws a book at the offender and runs through the doors into the adjacent smoking area, screaming: “He has a gun!” The offender slowly follows her and shouts, “Yes, I have a gun!” before inflicting another female student with a fatal shot. He takes a final shot at a salt and pepper grinder on a table near a professor and three female students before leaving the cafeteria and walking down the hallway. He passes a woman who appears to be unaware of the ongoing attack, walks into the toilet room and enters the middle booth. He locks the door, sits down, balances the shotgun on the toilet seat while pointing the barrel at his own head, fires, and dies instantly. Police officers later find his body along with 15 unused cartridges. Two died in the attack, a third was severely injured, and a fourth superficially wounded. All victims were female students.

### Grievances

Reviewing our closed and open-source material, we identify two simultaneously present grievances: an *academic* grievance toward Aarhus University as an institution based on failed attempts to achieve academic excellence and a *romantic* grievance toward women stemming from experiences of intimate rejection.

The academic grievance represents the common perspective on the Aarhus University shooting, namely that it was an act of violence that fits the lone-actor school shooter category. This is most obviously evidenced by the location of the attack, namely an educational institution, and the fact that the offender targeted students. Even though the police did not find any signs that the offender held a general hatred toward the university (Danish National Archives, 1994g), some evidence indicates otherwise. For instance, a member of staff at the Department of Nordic Studies describes how the offender wandered the office halls looking for his thesis supervisor shortly before the attack (Danish National Archives, 1994b; Bekholm and Hansen, 2019). He also says that 1 day, several months before the attack, the offender showed up at the supervisor’s office and almost jumped into the room, pointing at the supervisor with his fingers and making noises like he was firing a machine gun. According to the offender, this was meant as a practical joke, but in light of the future attack, this episode has become a source of speculation as to whether the supervisor also was an – and perhaps *the* – intended target. The offender constantly questioned the feedback he got on his thesis, and he had already changed supervisor twice because of disagreements. During an investigative interview, the supervisor says that he was under the impression that the offender slowly was beginning to realize that he would not be able to complete his thesis and obtain his degree (Danish National Archives, 1994b). On this account, committing an attack on university grounds might be conceived as retribution against an institution that did not support but rather, at least from the offender’s perspective, prohibited him from succeeding academically.

In addition to this academic grievance, we identify a romantic grievance that is more closely related to the misogynist worldview of the incel lone-actor than the school shooter. Hence, in contrast with common perspectives on the attack, we argue that the female victims were targeted not because they were *students* but because they were *women*. All the victims were female, and besides the professor sitting at a table with female students, there are no indications that the offender attempted to attack men. Furthermore, in a diary entry from November 1992, approximately 18 months before the attack, the offender writes: “When someone reads this, I have hopefully succeeded in killing some worthless bitches who will pay for the scars I carry” (Fisker, 2003; Bekholm and Hansen, 2019). In light of such writings, the outburst “I hate you!” prior to the first shot may indeed have been directed toward women in a general sense rather than the specific female victims, especially since there were no indications that the offender had any personal ties to any of the victims (Danish National Archives, 1994g).

As the offender indeed seems to have been motivated by two simultaneously present grievances, it becomes clear that the attack should neither be classified as a school shooting nor as an incel attack, as either category reduces the complexity of the offender’s motivations. Rather, we argue that the offense was a case of lone-actor grievance-fueled violence involving multi-facetted offender motivation.

### Childhood, adolescence, and university years

The offender was born in a Danish village in 1958 (Bekholm and Hansen, 2019). His father worked as a janitor, and his mother was a stay-at-home-mom (Fisker, 2003). When he was 9 years old, the family moved to a community housing area, and 1 year later, the offender’s younger brother was born (Bekholm and Hansen, 2019). Both boys grew up in severe poverty; the home was shabby, the wallpaper worn out, the boys slept on mattresses on the floor, and the furniture was made of spare bricks and wooden planks. Neither of the parents tended to the children’s mental or physical well-being, and the boys were often sent to school without a proper shower (Fisker, 2003).

While we still do not know exactly how specific grievances form (Silver et al., 2019), we tentatively propose that the grievances of the offender behind the Aarhus University shooting might stem from a life of academic as well as romantic hardships. The former seems to have persisted throughout most of the offender’s life. His former school principal and teachers vaguely remember him as a regular pupil who did not stand out in any way (Bekholm and Hansen, 2019); he had poor academic skills resulting in low grades, and sometimes one or two of the teachers would highlight his homework in class as an example of what *not* to do (Fisker, 2003). After finishing primary and high school, the offender served his military conscription and then did blue-collar labor for a couple of years (Fisker, 2003; Bekholm and Hansen, 2019). He started his educational program at Aarhus University at the age of 27, which also seems to have been a setting for academic bullying and humiliation. Again, a teacher highlights his home assignments as prime examples of poor work, and other students call him “the perpetual student” to make fun of how much time he spent completing his studies because of academic difficulties. This was exemplified by the final thesis writing process, which had dragged out over several years and involved multiple supervisors. Although he had written almost a 1,000 pages, the thesis was not at all ready for submission at the time of the attack.

Likewise, the offender seems to have experienced long-term marginalization and romantic rejection. His former teachers say, that in primary school, he was thin, pale, and never really caught the attention of girls. He often walked around with his eyes to the floor as if he was ashamed or wanted to hide from the world. Sometimes he acted like a fool in class in a desperate attempt to get attention, but no one seemed to like him or notice him. The younger brother says that about 13 years before the attack, the offender had a girlfriend for 2–3 years, and after the break-up he never talked about girls again (Danish National Archives, 1994c). A former friend tells that he was under the impression that girls did not even interest the offender, and that he did not appear to have had any female acquaintances during the last 7 years (Danish National Archives, 1994f). It seems, however, that this was more because of lack of success than lack of trying. A female employee in the local supermarket near the offender’s home describes how he once asked whether she wanted to come to his apartment after work, and that he could exhibit socially inappropriate dating behavior in public settings (Bekholm and Hansen, 2019). Likewise, people living in his dorm describe how he once had a falling out with one of the girls in the common room. Moreover, even though he lived at a very social dorm, he almost exclusively kept to himself, he only attended one social event and he left early (Bekholm and Hansen, 2019). Two former dorm mates describe him as a person who seemed to be uncomfortable with social interaction, avoided eye contact, and was difficult to start a conversation with. His last thesis supervisor says that he was under the impression that the offender had no social acquaintances at university, and that he generally was very socially isolated and lonely (Danish National Archives, 1994b). He cut ties to his parents, ceased contact with a friend about 2 years before the attack, and did not seem to spend time with anyone except his younger brother, who says that it was his clear impression that the offender grew more and more socially aversive (Bekholm and Hansen, 2019).

Based on the above, we contend that the identified academic and romantic grievances might have their root in a life of personal hardships, i.e., educational struggles and difficulties with social interaction, especially when it comes to attracting a romantic partner. Moreover, we tentatively propose that these experiences might have been a key factor in the development of a personality structure characterized by grandiose entitlement, which we elaborate below.

### Grandiose narcissistic entitlement

As mentioned, a factor that may render individuals particularly prone to react violently against perceived injustice and humiliation is the presence of narcissistic traits or grandiose entitlement (Bondu and Scheithauer, 2015; Gøtzsche-Astrup and Lindekilde, 2019; Storr, 2021). Based on the data at hand, there are several indications that the perpetrator of the Aarhus University shooting was characterized by such a personality structure.

For starters, the younger brother says that the reason the offender started an educational program at the university was that he wanted to do something more “prestigious” than blue-collar work: “It was like, well, now that you went to university, you had to be fancy and all – because now you were above others” (Fisker, 2003, 11:20–11:29). People living at the dorm and other acquaintances tell that the offender tried to make others believe that companies desperately wanted employees “with an education like his” and that he often pretended to be extremely clever, though no one really believed him (Danish National Archives, 1994f). In an attempt to underline his “academic superiority,” he would often make fun of and mock his fellow students during lectures and classes when giving feedback on their homework. He also changed his appearance to match his new lifestyle and academic ambitions (Bekholm and Hansen, 2019). He always wore a tie and bought clothes “suitable for an academic.” In a letter to his thesis supervisor, he said: “I have chosen to write my thesis using a fountain pen (Daniel Hechter) as I do not want to do it on a typewriter. Surely, you can appreciate that I keep the art of handwriting alive” (Danish National Archives, 1994j, original underlining). In general, he seemed very concerned about his appearance and what others thought about him, not only at the dorm or university but also in public. A former employee at his local video store says that the offender insisted on bringing his own picture for a membership card and apologized for only wearing a polyester tie, because he “usually only wore silk ties” (Fisker, 2003).

Indeed, a lot of the offender’s life choices and behavior, especially during the university years, seem to be driven by a strong desire for status and fame, possibly in an attempt to symbolically distance himself from his childhood and adolescence, which appear to have been the exact opposite.

### Violent rumination

Besides the risk factors and mechanisms of radicalization already accounted for, much evidence indicates that the offender often fantasized about mass violence before committing the attack at Aarhus University. According to his younger brother, the offender developed a profound interest in mass murderers after watching a movie about it. He read books on the subject and started collecting news articles about murderers and killers. “Everything about mass murder, whether on TV or in newspapers, he swallowed whole, and he could spend an entire Sunday reading about murders in the paper” (Bekholm and Hansen, 2019). In addition, the brother says that the offender easily could define a mass murder and knew a lot about different murderers, whom they had killed, and how they had done it (Danish National Archives, 1994c). The brother also describes how the two of them sometimes joked about killing people and becoming mass murderers, and one time the offender suggested that they should “go down to the common room [at the offender’s dorm] and do some killing” (Danish National Archives, 1994c) and that he “wanted to be the very first mass murderer in Denmark” (Danish National Archives, 1994c; Fisker, 2003; Bekholm and Hansen, 2019). The offender also said that in contrast to some very infamous mass murderers who cut off their victim’s skin or the like, we would not do so; if he was to kill, he “wanted to see blood” (Danish National Archives, 1994i). During the search of his apartment, police investigators found a personal diary in which the offender, based on newspaper articles and other media news, noted how many people had either been killed or died every day during the last couple of months before the attack at Aarhus University (Danish National Archives, 1994e).

### Mental health and strain

In close proximity to the attack, the offender experienced severe issues regarding mental health and strain due to negative life events. In a letter written approximately 2 years before the attack, he writes: “I am unable to get my aggressions out, and I have so many emotional scars that I mostly just feel dead and empty inside. Recently, I have realized that I will never be rid of my problems, and it would have been best to end it all at least 10 years ago” (Fisker, 2003). Some months before the attack, the offender started seeing a psychiatrist (Bekholm and Hansen, 2019). Our data does not indicate what was discussed in therapy, but both a diary entry and a prescription from the psychiatrist reveal that the offender started taking anti-depressives (Fisker, 2003). However, the offender suddenly stopped seeing the psychiatrist 1 month before the attack at Aarhus University, indicating that his mental state only worsened in the time before his violent actions.

In addition to deteriorating mental health, the offender experienced negative life events around the time of the attack. As mentioned, his thesis supervisor made the presumption that the offender was aware that he would not be able to complete his studies and get his university degree. Apparently, the state and local municipality shared this view and withdrew his social benefits, leaving him with a debt of DKK 200,000 (approximately £22,222), the prospect of not being able to pay his rent, no university degree, and hence no job in sight (Bekholm and Hansen, 2019). It is our contention that these factors in combination may have contributed to putting the offender in a state of severe desperation or distress where mass violence was seen as a potential “last resort” (e.g., Meloy and Gill, 2016). These factors may have accelerated violent radicalization and been important in explaining the timing of the attack.

### Summary

Our analysis shows that the radicalization trajectory of the offender behind the Aarhus University shooting was characterized by a mix of (1) multiple grievances, (2) deprived social background, (3) varieties of interpersonal rejection in childhood, adolescence, and adulthood, (4) a grandiose narcissistic personality structure, (5) rumination on violent fantasies, (6) and issues regarding mental health and strain. These are all characteristics generally associated with lone-actor radicalization (e.g., Kenyon et al., 2021; Ebbrecht, 2022). So far, our findings are thus in line with the principle of equifinality, as the offender’s radicalization trajectory indeed seems to have been a multifactorial process.

However, a central caveat of our study is that the data available does not allow us to determine the relative causal role of, e.g., the normal psychological functioning at play, and the indications of psychopathological depression seems to develop in the months before the attack. It is a general finding that mental health problems are more prevalent among lone actors than group-based perpetrators of terrorism or mass violence (e.g., Gruenewald et al., 2013). For instance, in their study on 119 lone-actor terrorist and 448 group-based actors, Corner and Gill (2015) found that lone actors were 13.5 times more likely to have a history of mental illness. Likewise, Allely et al. (2017) found tentative evidence of autism-spectrum disorder in six out of 75 cases of mass shootings, which is a prevalence rate about eight times higher compared to the general population. However, while mental health issues often occur in the case of lone-actor violence, it seems that there are about just at many cases where they are absent. In their systematic review on mental health and violent extremism, Gill et al. (2020) finds that confirmed diagnoses never exceed 45% in samples on lone-actor terrorists. Similarly, Ebbrecht (2022) conducted a systematic review on risk factors and mechanisms of radicalization among lone-actor terrorists, workplace attackers, school shooters, rampage shooters and violent Incels, where most studies reported that 40–50% of the offenders in their respective samples had mental health problems. Hence, psychopathology does not seem necessary for lone-actor violence to occur, and following the principles of equifinality and multifinality, the role of psychopathology gets more complicated as its mere presence does not equal relevance in lone-actor radicalization (Al-Attar, 2020a, 2020b). To quote Gill et al. (2020, p.68): “Where present, it [mental health problems, red.] might be a driving force, it might inflame other stressors and have snowball-effect, it might be a by-product of violent extremism behaviors, or it might be playing no role whatsoever.”

## Discussion

The Aarhus University shooting has been described in journalistic writing and has gone down in Danish public memory as the only Danish school shooting in history. Is this an accurate categorization of the case? Does the implied motivation of revenge for personal experiences of school bullying and academic humiliation capture the grievances that led the perpetrator to commit his attack? As it should be clear by now, we believe that the answer to these questions is no. Our case study clearly shows that the offender held grudges against the university, fellow students and supervisors. But it also clearly shows that he experienced other grievances related to general social incompetence, which particularly led to limited interaction with women and intimate rejections. Combined with a personality of grandiose and narcissistic entitlement partly shaped by experiences of hardship and deprivation in childhood and adolescence, this fueled a hatred against the university and women and a desire for revenge. Only when we take this complex motivational impetus into account can we make full sense of the choice of target: women at the university.

Rather than simply recasting the Aarhus University shooting as an incel attack considering the evidence of the offender’s misogynistic views, we believe the case exemplifies the need to embrace the conceptualization of lone-actor grievance-fueled violence, which points toward the common genesis of lone-actor violence and allows for multi-faceted offender motivations. Used as a prism, the case highlights three pitfalls of relying on a typological approach to lone-actor violence; pitfalls, we argue that the application of a lone-actor grievance-fueled violence framework can help us avoid. First, the available categories for lone-actor violence depend on context- and time-sensitive understandings of motivations. Although the police in the case of the Aarhus University shooting was clearly aware of the offender’s romantic grievances, these were never really brought forward as part of the motive in subsequent writing about the case. Why? We argue that this was partly because the ideology of inceldom and the category of lone-actor incel violence had not been established and defined at the time of the attack. Romantic grievances were obviously known at the time as motivations for murders when the offender knew the victim but not as the motivational impetus for lone-actor mass violence. In contrast, the offender’s academic grievances were recognizable and matched public conceptions of the school shooter. We are convinced that had the attack happened today after infamous incel attacks and the public debate about the incel online subculture, the romantic grievances and signs of extreme misogyny would have received more attention and potentially led the case to be categorized as incel lone-actor violence rather than a school shooting. To illustrate, incel violence is now explicitly mentioned in “Assessment of the Terrorist threat to Denmark” (Centre for Terror Analysis, 2022), and the incel terminology was much more predominant in a recent Danish trial against a 27-year-old man who was convicted for planning multiple school shootings in Denmark. While the attack planning is still conceptualized as a school shooting, the offender’s misogynist worldview and online activity have explicitly been linked to the incel category (Hamilton, 2022). As such, a typological approach to lone-actor violence seems to concur with the social constructionist point that how one comprehends the social world is dependent on the knowledge (categories) available at a given time in history (Hacking, 1995), even though the motivational impetus might remain unchanged.

Second, we contend that a typological approach to lone-actor violence led to reductionism and loss of motivational complexity. In trying to neatly and mutually exclusively categorize cases, the typological approach risks making observers blind to the complexity of multiple and intertwined motivations. Lone-actor terrorists may hold both personal and political grievances, just as school shooters may hold both romantic and academic grievances. In fact, whenever we have sufficiently detailed data to detect this, such multi-facetted motivations turn out to be the norm rather than a “fringe case” that challenges our conceptual map. The motivational impetus for lone-actor violence is more often than not “messy.”

Third, the typological approach to understanding and investigating lone-actor violence leads to theoretical and conceptual multiplication rather than coherence. In the academic literature, scholars invested in the study of one particular category of lone-actor violence tend to theorize this type of violence anew and with a special view to factors that make the category of cases distinct rather than to commonalities with other types of lone-actor violence. We argue that rather than leading to an ever more refined and nuanced understanding of lone-actor violence, this tendency undermines attempts at comprehensive theorization and conceptual clarity. This is a challenge to scientific advancement in the field and to, e.g., police work, which often relies on academic conceptions to interpret cases.

Based on the outlined pitfalls of the typological approach, we advocate for moving away from such a categorization of violent lone-actor types and toward the unifying concept of lone-actor grievance-fueled violence. By focusing on grievances as drivers of lone-actor violence, this approach makes assessment of motivation less dependent on context- and time-dependent categories and allows for multiple grievances to coexist simultaneously. As indicated, we believe this approach has practical implications as well as academic merit by unifying otherwise separate strands of literature and theory. Most importantly, the lone-actor grievance-fueled violence framework has implications for risk assessments. Multifaceted motivations call for holistic risk assessments. If we want to identify risks of lone-actor grievance-fueled violence in time to intervene, actors that may hold different pieces of the puzzle need to come together. In this sense, a move toward the concept of lone-actor grievance-fueled violence translates into a need for inter-agency collaboration around risk assessment on the ground. For example, schoolteachers still need to be mindful of student experiences of bullying and academic humiliation turning into violent grudges, and others may inform schoolteachers that a student of concern is airing, e.g., political grievances on social media or frequenting incel online fora. This is only possible if cases of concern are risk assessed by inter-agency teams representing multiple professional backgrounds. In line with this argument, the move toward the unifying concept of lone-actor grievance-fueled violence accentuates the problem of too narrow policies and action plans aimed at countering violent extremism, online misogyny or school shootings. When we begin to conceive the problem in a more holistic and unified manner, the solution also appears to be interconnected. While narrow policies focusing on, e.g., prevention of school shootings are important in their own right, addressing the problem at a higher level of abstraction may be more efficient in the long run.

## Conclusion

The scientific community, police intelligence services, and the media tend to categorize violent lone actors within a typological framework. Using the Aarhus University shooting as a prism, we have shown that such categorization simplifies our understanding of offender motivation. We recommend turning away from a categorical approach to lone-actor violence and toward the unifying conception of lone-actor grievance-fueled violence instead. Using detailed, restricted material, we show that the offender was motivated simultaneously by academic and romantic grievances, leading him to attack female students at the university. While both sets of grievances are clearly visible in the case files and noticed by the investigating police officers, the attack has been canonized as the only Danish school shooting. For example, Wikipedia pages do not mention potential misogynist motivations. We argue that this is the product of a typological approach to understanding lone-actor violence that leads to a reduction of complex motivations and an inability to look beyond the available and limited categories of the time. Furthermore, we argue that the categorical approach to lone-actor violence leads to theoretical and conceptual multiplication, inhibiting scientific advancement in the field. We are not arguing that a turn toward a unifying conception of lone-actor grievance-fueled violence will lead to immediate theoretical gains or practical advantages, but that such a turn is a prerequisite for advancement in the longer run.

## Data availability statement

The data analyzed in this study is subject to the following licenses/restrictions: Access to restricted police files is granted by Danish authorities. Requests to access these datasets should be directed to Danish National Archives (mailbox@sa.dk).

## Ethics statement

Ethical review and approval was not required for the study on human participants in accordance with the local legislation and institutional requirements. Written informed consent from the [patients/ participants OR patients/participants legal guardian/next of kin] was not required to participate in this study in accordance with the national legislation and the institutional requirements. Oral informed consent was obtained from the individual(s) for the publication of any potentially identifiable images or data included in this article.

## Author contributions

All authors listed have made a substantial, direct, and intellectual contribution to the work and approved it for publication.

## Funding

This research was supported by ERC Consolidator Grant (grant# 37480).

**Figure 1:**
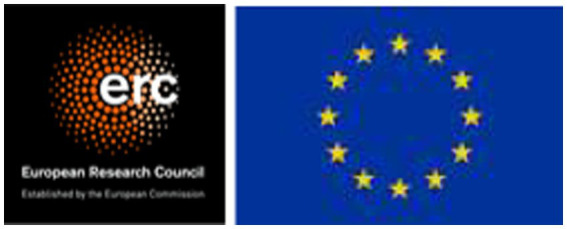


## Conflict of interest

The authors declare that the research was conducted in the absence of any commercial or financial relationships that could be construed as a potential conflict of interest.

## Author disclaimer

Views and opinions expressed are however those of the authors only and do not necessarily reflect those of the European Union or the European Research Council Executive Agency. Neither the European Union nor the granting authority can be held responsible for them.

## Publisher’s note

All claims expressed in this article are solely those of the authors and do not necessarily represent those of their affiliated organizations, or those of the publisher, the editors and the reviewers. Any product that may be evaluated in this article, or claim that may be made by its manufacturer, is not guaranteed or endorsed by the publisher.
